# Proceedings of the Ohio State Society

**Published:** 1883-04

**Authors:** 


### The Proceedings of the Homœopathic Medical Society of Ohio, Eighteenth Annual Session, held at Springfield, May 9th and 10th, 1882. H. E. Beebe, M. D., Secretary.

These proceedings are given in a neat volume of 263 pages, and contain very many interesting papers. We are glad to see so many of them are on practical subjects.

Writing on post-partum hæmorrhage, Dr. Eaton thinks it very foolish to lose valuable time in seeking the *simillimum* for the case when ergot can be so readily administered. But suppose *ergot* does not *act*, how then, Professor? The Doctor says we must turn out the clots and give ergot. Of course, the first and most imperative duty is to "turn out the clots," and also to compress the uterus, but sometimes in a weak, relaxed patient these measures fail. It is therefore better to always give the *simillimum*, and also to assist mechanically.

A story is told about the late Professor Charles D. Meigs, who, when lecturing years ago at the Jefferson Medical School, in this city, on post-partum hæmorrhage, walked the rostrum for a half hour repeating, "turn out the clots," "turn out the clots," and then dismissed the class without further lecture, wishing, he said, to impress that one idea upon them.

We are sorry space does not allow our noticing many of the valuable papers contained in these proceedings.

